# Exosome-mediated delivery of CRISPR/Cas9 for targeting of oncogenic Kras^G12D^ in pancreatic cancer

**DOI:** 10.26508/lsa.202000875

**Published:** 2021-07-19

**Authors:** Kathleen M McAndrews, Fei Xiao, Antonios Chronopoulos, Valerie S LeBleu, Fernanda G Kugeratski, Raghu Kalluri

**Affiliations:** 1 Department of Cancer Biology, Metastasis Research Center, University of Texas MD Anderson Cancer Center, Houston, TX, USA; 2 Department of Bioengineering, Rice University, Houston, TX, USA; 3 Department of Molecular and Cellular Biology, Baylor College of Medicine, Houston, TX, USA; 4 Feinberg School of Medicine, Northwestern University, Chicago, IL, USA

## Abstract

CRISPR/Cas9 is a promising technology for gene editing. To date, intracellular delivery vehicles for CRISPR/Cas9 are limited by issues of immunogenicity, restricted packaging capacity, and low tolerance. Here, we report an alternative, nonviral delivery system for CRISPR/Cas9 based on engineered exosomes. We show that non-autologous exosomes can encapsulate CRISPR/Cas9 plasmid DNA via commonly available transfection reagents and can be delivered to recipient cancer cells to induce targeted gene deletion. As a proof-of-principle, we demonstrate that exosomes loaded with CRISPR/Cas9 can target the mutant *Kras*^*G12D*^ oncogenic allele in pancreatic cancer cells to suppress proliferation and inhibit tumor growth in syngeneic subcutaneous and orthotopic models of pancreatic cancer. Exosomes may thus be a promising delivery platform for CRISPR/Cas9 gene editing for targeted therapies.

## Abstract (precis)

This work identifies the use of exosomes to specifically deliver CRISPR/Cas9 to target oncogenic KrasG12D mutation in pancreatic cancer as a nonviral therapeutic strategy.

## Introduction

The CRISPR/Cas9 system, originally found in nature as a prokaryotic adaptive immune system, has been repurposed into a powerful tool in genome engineering, providing a versatile programmable platform for precise gene editing (1). CRISPR/Cas9 is a two-component system consisting of Cas9, an RNA-guided endonuclease capable of cleaving double-stranded DNA, and a 20-nucleotide-long synthetic guide RNA (sgRNA) that is engineered to program the sequence specificity of Cas9 for DNA cleavage. By delivering the Cas9 nuclease complexed with a sgRNA into a cell, the cell’s genome can be cut at a desired location, allowing existing genes to be removed or edited in vivo. Gene knockouts are driven by the formation of Cas9-induced insertions-deletions (indels) that disrupt the open reading frame of a target gene rendering it non-functional (2). Over the years, efforts have been aimed at harnessing CRISPR gene editing for developing therapeutic interventions for monogenic diseases, as well as more complex multifactorial diseases such as cancer (3).

Despite significant advances in the field, a major bottleneck in unlocking the enormous translational potential of CRISPR/Cas9 for in vivo gene therapy remains the choice of an appropriate delivery vehicle. An ideal vector would be safe, stable, non-immunogenic and highly efficient in delivering the CRISPR/Cas9 payload while retaining targeting specificity and minimizing off-target activity. Both viral and nonviral vectors have gained popularity in recent years (4, 5, 6). Cas9 and sgRNA are typically introduced into recipient cells either in the form of plasmid DNA, in vitro transcribed (IVT) mRNA, or protein in the form of a pre-assembled RNP complex. Viral vectors such as adeno-associated viruses have been the leading tool for in vivo CRISPR/Cas9 delivery but are limited by issues related to restricted packaging capacity (<5 kb), neutralizing antibodies against adeno-associated virus capsids, potential immunogenicity and insertional mutagenesis raising concerns about safety in clinical practice (7, 8, 9). Nonviral carriers, such as synthetic liposomes or polymeric and metallic nanoparticles are well characterized, do not rely on a viral genome, and are tunable through chemical modifications (10). Limitations associated with synthetic nanoparticles include accelerated blood clearance, low efficiency, problematic biocompatibility, toxicity/immunogenicity, and potential issues with therapeutic cargo release (10).

Exosomes represent a promising alternative delivery platform for CRISPR/Cas9 gene therapy that circumvents many of the limitations associated with viral and nonviral vectors. Exosomes are a subtype of nanoscale membranous vesicles naturally released from the endocytic compartment of all live cells and carry molecular cargo (DNA, RNA, protein, and lipids) reflective of their cell-of-origin (11). In contrast to synthetic nanoparticle carriers, exosomes are typically immunologically inert and non-cytotoxic if purified from a compatible cell source (12). Unlike liposomes, exosomes carry various transmembrane and membrane-anchored proteins that extend their half-life in blood circulation by evading phagocytic clearance while conferring superior cellular uptake and subsequent delivery of their internal cargo to recipient cells (13). Previous work from our laboratory has highlighted the potential of engineered exosomes (iExosomes) in RNAi delivery (siRNA/shRNA) to target oncogenic *Kras*^*G12D*^ in pancreatic cancer (13). Exosomes for CRISPR/Cas delivery has been reported for the knockout of PARP-1 in ovarian cancer (14). Exosomes also have the ability to cross multiple biological barriers and can be engineered to encapsulate CRISPR/Cas9 cargo as plasmid DNA or in a more transient format, namely, mRNA and RNP to avoid risks associated with sustained expression of bacterial Cas9 (14,15,16).

Here, we sought to investigate whether exosomes can function as natural cell-derived nanocarriers for CRISPR/Cas9 gene editing therapy. Our study provides proof-of-concept that exosomes can be successfully engineered to encapsulate and deliver CRISPR/Cas9 plasmid DNA to knockout the mutant *Kras*^*G12D*^ oncogenic allele in pancreatic cancer cells leading to inhibited proliferation and suppressed tumor growth in vivo.

## Results

### CRISPR/Cas9-guided gene editing for targeting oncogenic *Kras*^*G12D*^ in pancreatic cancer cells

**Figure S1.**
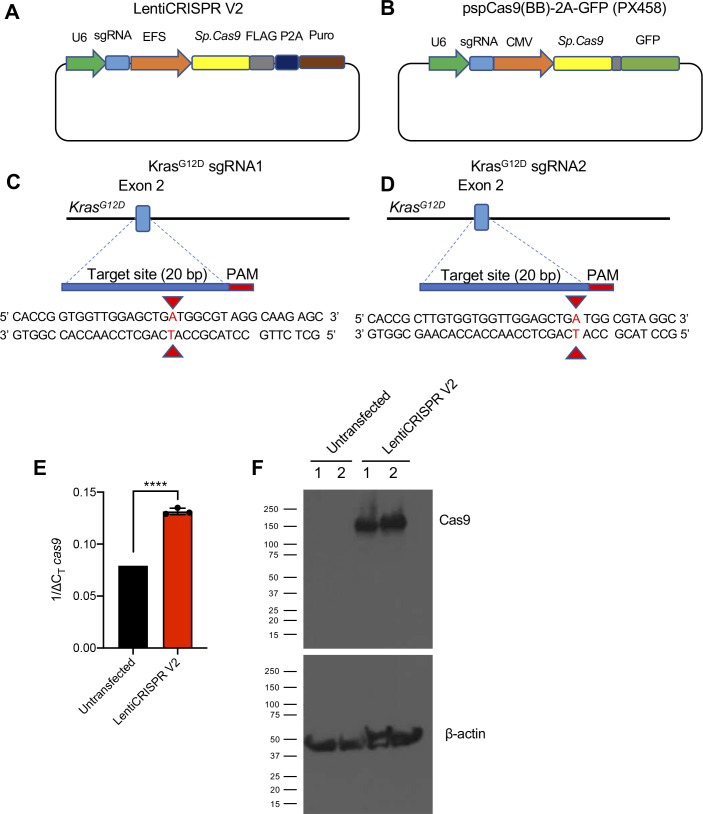
CRISPR/Cas9 vectors to target the murine genomic locus of *Kras*^*G12D*^. **(A, B)** Plasmid map of the Cas9/synthetic guide RNA (sgRNA) expression vector LentiCRISPR v2 and (B) PX458. **(C, D)** Sequence of sgRNA1 (C) and sgRNA2 (D) targeting the murine *Kras*^*G12D*^ allele. **(E, F)** Cas9-overexpressing KPC689 cells were generated by transfection with LentiCRISPR V2 with subsequent puromycin selection and clonal expansion. **(E)** *cas9* mRNA expression levels were tested with quantitative PCR. Data are normalized to *18s* levels and are presented as presented as mean ± standard deviation. Unpaired *t* test performed. **(F)** Cas9 protein expression was validated with Western blotting. Loading control: β-actin. ***P < 0.001. Source data are available for this figure.

To deliver Cas9/sgRNA into cells, we used two commercially available Cas9-encoding plasmids: LentiCRISPR V2 (lentiviral backbone) and pSpCas9(BB)-2A-GFP (PX458). One of two custom sgRNAs targeting the murine genomic locus of oncogenic *Kras*^*G12D*^ (sgRNA1 and sgRNA2) was inserted into the backbone of the two Cas9-encoding plasmids using standard restriction-ligation cloning (Fig S1A–D). The CRISPR/Cas9 encoding plasmids were subsequently delivered via lipofectamine transfection into cultured murine pancreatic cancer cells isolated from the autochthonous pancreatic cancer model *Pdx1-Cre*; *LSL-Kras*^*G12D/*+^; *LSL-Trp53*^*R172H/*+^ (KPC) harboring an oncogenic *Kras*^*G12D*^ mutation (KPC689) (17).

**Figure 1.**
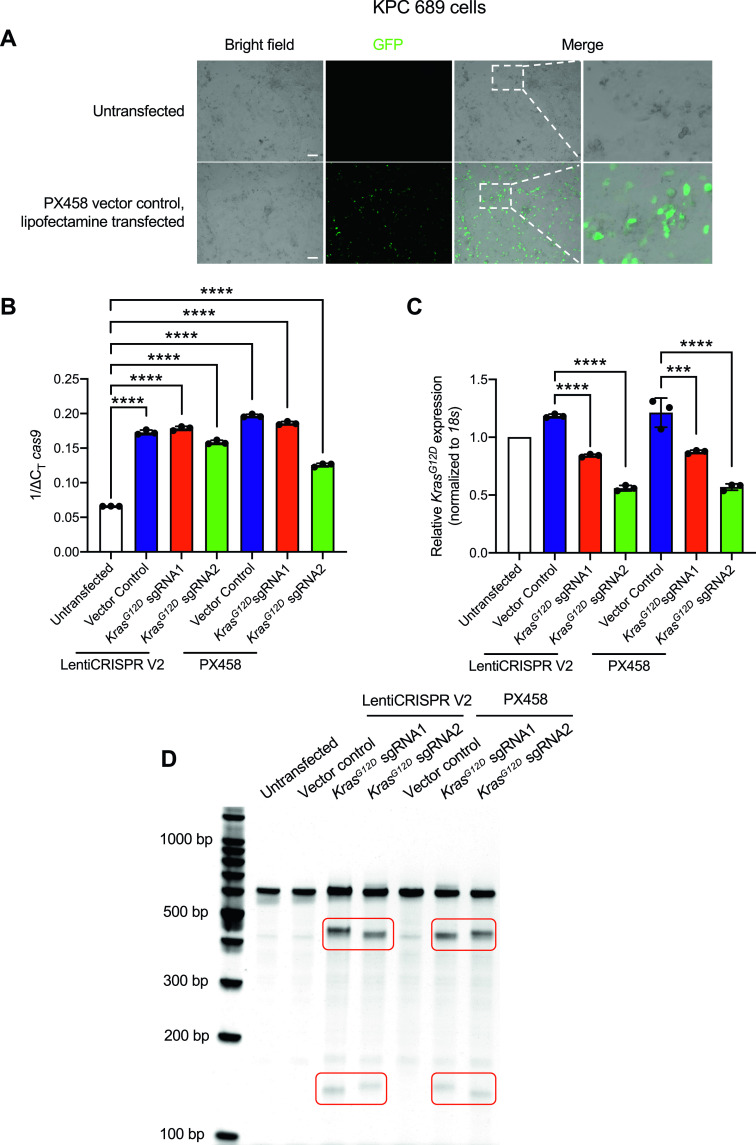
CRISPR/Cas9–mediated gene editing suppresses oncogenic *Kras*^*G12D*^ in vitro. KPC689 cells were transfected with 5 μg plasmid DNA (Cas9/Kras^G12D^ gRNA1/2 with LentiCRISPR V2 or PX458 backbone, and the Cas9 vector controls) by Lipofectamine 2000 for 48 h. **(A)** Epifluorescence microscopy imaging was used to evaluate transfection efficiency of lipofectamine 2000 by using GFP/Cas9 vector control (PX458) plasmid. Scale bar, 100 μm. **(B, C)** Quantitative PCR was used to evaluate mRNA expression levels of *cas9* (B) and *Kras*^*G12D*^ (C). **(C)** Data in (C) are normalized to *18s* and untransfected control. One-way ANOVA with Tukey’s multiple comparisons test was used to evaluate mean differences among groups based on ΔC_T_ values. **(D)** T7/Surveyor assay was used to evaluate gene editing in genomic DNA of KPC689 cells following transfection with Lipofectamine after 48 h. All results are expressed as mean ± standard deviation. ***P < 0.001, ****P < 0.0001. Source data are available for this figure.

Epi-fluorescence imaging and quantitative PCR (qPCR) were performed to evaluate the delivery and transfection efficiency with CRISPR/Cas9–encoded plasmid DNA. KPC689 cells transfected with the PX458-Cas9 empty vector showed robust GFP expression after 48 h (Fig 1A). At the transcript level, de novo expression of Cas9 mRNA was observed in KPC689 cells after transfection with all Cas9/sgRNA plasmids including the Cas9 vector controls, as well as the respective Cas9/sgRNA co-expressing plasmid vectors (Fig 1B). Stable transfection with LentiCRISPR V2 resulted in robust Cas9 expression on the mRNA and protein level (Fig S1E and F).

Next, we assessed the presence of gene editing and relative efficacy of targeted gene disruption of oncogenic *Kras*^*G12D*^ in KPC689 cells. CRISPR/Cas9–mediated gene targeting resulted in significant suppression of *Kras*^*G12D*^ mRNA expression in KPC689 cells after transfection with all Cas9/sgRNA co-expressing plasmids for either LentiCRISPR V2 or PX458, whereas *Kras*^*G12D*^ sgRNA2 showed the largest suppression of *Kras*^*G12D*^ mRNA (Fig 1C). Double stranded breaks in DNA are repaired via the non-homologous end joining (NHEJ) pathway resulting in small indels that are typically detectable with DNA mismatch cleavage detection assays. To confirm gene editing at the DNA level, a mismatch T7/surveyor assay was used. Successful Cas9-mediated cleavage and gene editing were confirmed for the KPC689 cells after transfection with the Cas9/sgRNA co-expressing plasmids but not with the Cas9 vector controls (Fig 1D). Taken together, lipofectamine-based cellular transfection is successful in delivering Cas9/sgRNA-encoded plasmid vectors into cultured pancreatic cancer cells for targeted CRISPR-mediated knockdown of oncogenic *Kras*^*G12D*^ in vitro.

### Exosome-mediated delivery of CRISPR/Cas9 plasmid DNA disrupts oncogenic *Kras*^*G12D*^ and suppresses proliferation in pancreatic cancer cells

**Figure 2.**
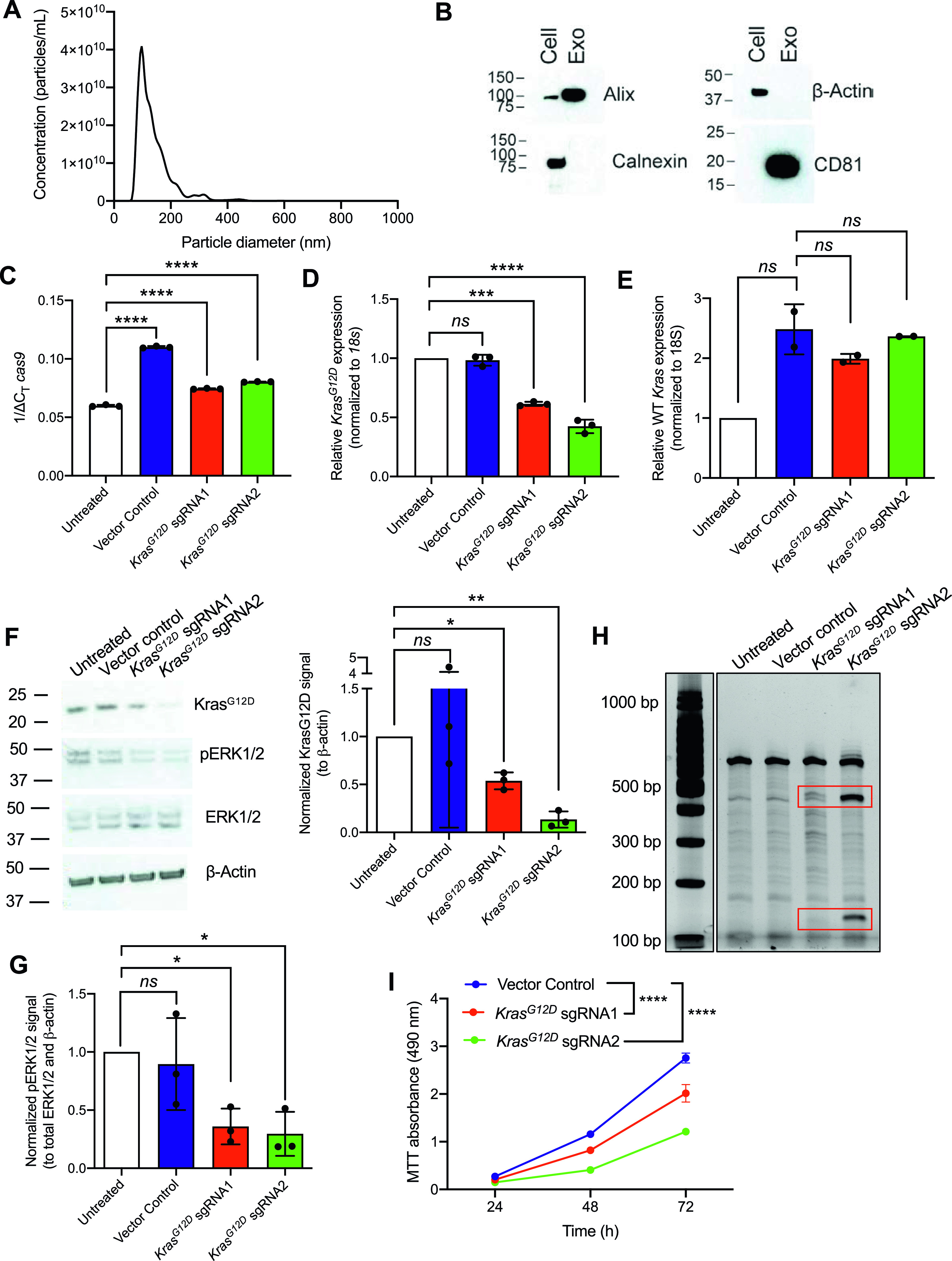
Exosome-mediated delivery of CRISPR/Cas9 disrupts oncogenic *Kras*^*G12D*^ in vitro and inhibits proliferation. **(A)** Representative size distribution and concentration of HEK293T exosomes measured by nanoparticle tracking analysis. **(B)** Representative Western blot for exosome markers Alix and CD81 and exclusion markers calnexin and β-actin. Cell, HEK293T cell lysate; Exo, HEK293T exosomes. **(C, D, E)** Quantitative PCR was used to evaluate mRNA expression levels of *cas9* (C), *Kras*^*G12D*^ (D), and WT *Kras* (E) of KPC689 cells treated with HEK293T exosomes (2,500 exosomes/cell) containing plasmid DNA (10 μg DNA/10^9^ exosomes) every day for 3 d. **(C)** Data in (C) are normalized to *18s*. **(D, E)** Data in (D, E) are normalized to *18s* and untransfected control. **(C, D)** One-way ANOVA with Tukey’s multiple comparisons test was used to evaluate mean differences among groups based on ΔC_T_ values for (C, D). **(E)** Brown–Forsythe ANOVA with Dunnett’s T3 multiple comparisons test was used to evaluate mean differences among groups based on ΔC_T_ values for (E). **(F)** Western blot for Kras^G12D^, pERK1/2, total ERK1/2, and β-actin (left panel) of KPC689 cells following treatment with exosomes containing CRISPR/Cas9 plasmid DNA. Quantification of Kras^G12D^ (normalized to β-actin) as assessed via Western blot (right panel). Data are normalized to untreated. One sample *t* test performed comparing each group to untreated. Full length blots are presented in Fig S3A. **(G)** Quantification of phospho-ERK1/2 (normalized to total ERK1/2 and β-actin) as assessed via Western blot (right panel). Data are normalized to untreated. One sample *t* test performed comparing each group to untreated. Full length blots are presented in Fig S3A. **(H)** T7/Surveyor assay was used to evaluate editing in genomic DNA of KPC689 cells. **(I)** MTT assay was used to evaluate cell viability/proliferation rates in KPC689 over the course of 72 h following treatment with exosomes loaded with CRISPR/Cas9 plasmid DNA. Two-way ANOVA with Tukey’s multiple comparison test was performed. Data are expressed as mean ± standard deviation. ***P < 0.001, ****P < 0.0001, *ns*, not significant. Source data are available for this figure.

**Figure S2.**
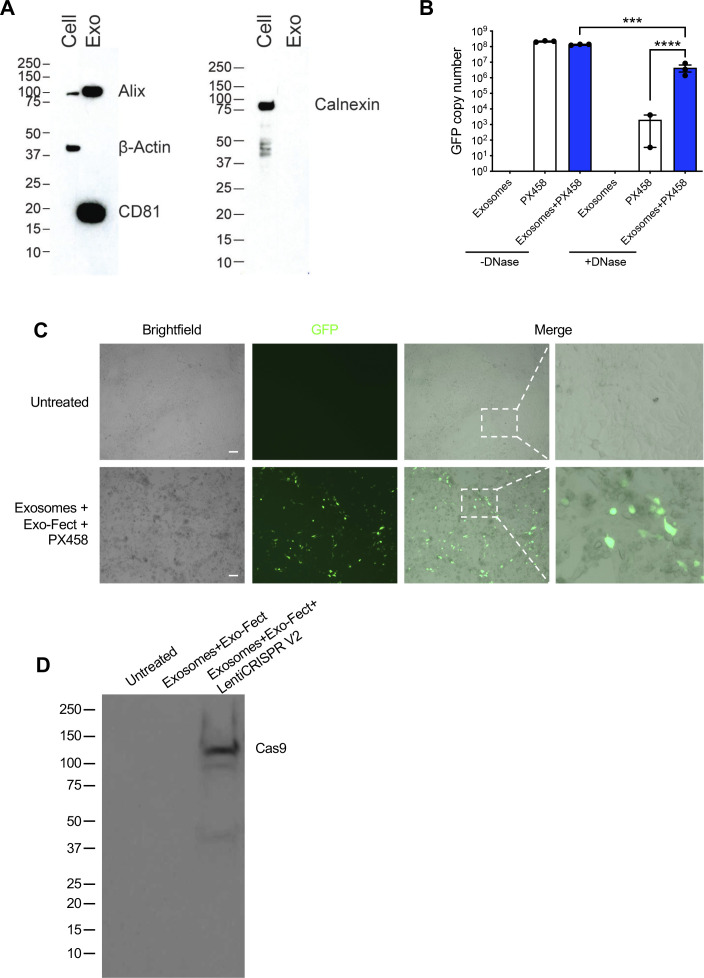
Uncropped Western blots of exclusion and exosomal markers and protein expression of GFP and Cas9 in cells treated with exosomes containing plasmid DNA. **(A)** Uncropped full blots of CD81, alix, calnexin, and β-actin. Blots correspond to the images displayed in Fig 2B. Cell, HEK293T cell lysate; Exo, HEK293T exosomes. **(B)** GFP copy number analysis of exosomes loaded with PX458 plasmid DNA. −DNase, no DNase treatment; +DNase, with DNase treatment. Data are presented as presented as mean ± standard deviation. **(C)** Images of KPC689 cells treated with exosomes containing PX458 plasmid. Scale bar, 100 μm. **(D)** Western blot for Cas9 protein in HEK293T cells treated with exosomes loaded with LentiCRISPR v2 plasmid. Source data are available for this figure.

Exosomes were purified from the culture supernatant of human embryonic kidney HEK293T epithelial cells by differential ultracentrifugation. The size distribution and presence of common exosomal markers, namely, Alix and the tetraspanin CD81, were validated with nanoparticle tracking analysis (NTA, Fig 2A) and Western blot (Figs 2B and S2A), respectively. Moreover, the commonly used exclusion markers calnexin and β-actin were detected in whole cell lysates of parental cells, but not in the exosomes (Figs 2B and S2A).

To examine the ability of HEK293T exosomes to function as delivery nanocarriers for the CRISPR gene editing machinery, we loaded CRISPR/Cas9 encoded plasmid vectors into HEK293T exosomes using the commercially available exosome transfection reagent Exo-Fect. We analyzed GFP copy number in exosomes loaded with PX458 plasmid DNA by qPCR and observed a reduction in copy number after DNase treatment (Fig S2B), indicating that a portion of loaded DNA is encapsulated within exosomes and is resistant to nucleases. KPC689 cells treated with exosomes containing PX458 plasmid DNA displayed expression of GFP (Fig S2C). Moreover, HEK293T cells treated with exosomes containing LentiCRISPR V2 expressed Cas9 at the protein level (Fig S2D). Together, these data indicate successful transfer and translation of plasmid DNA mediated by exosomes.

**Figure S3.**
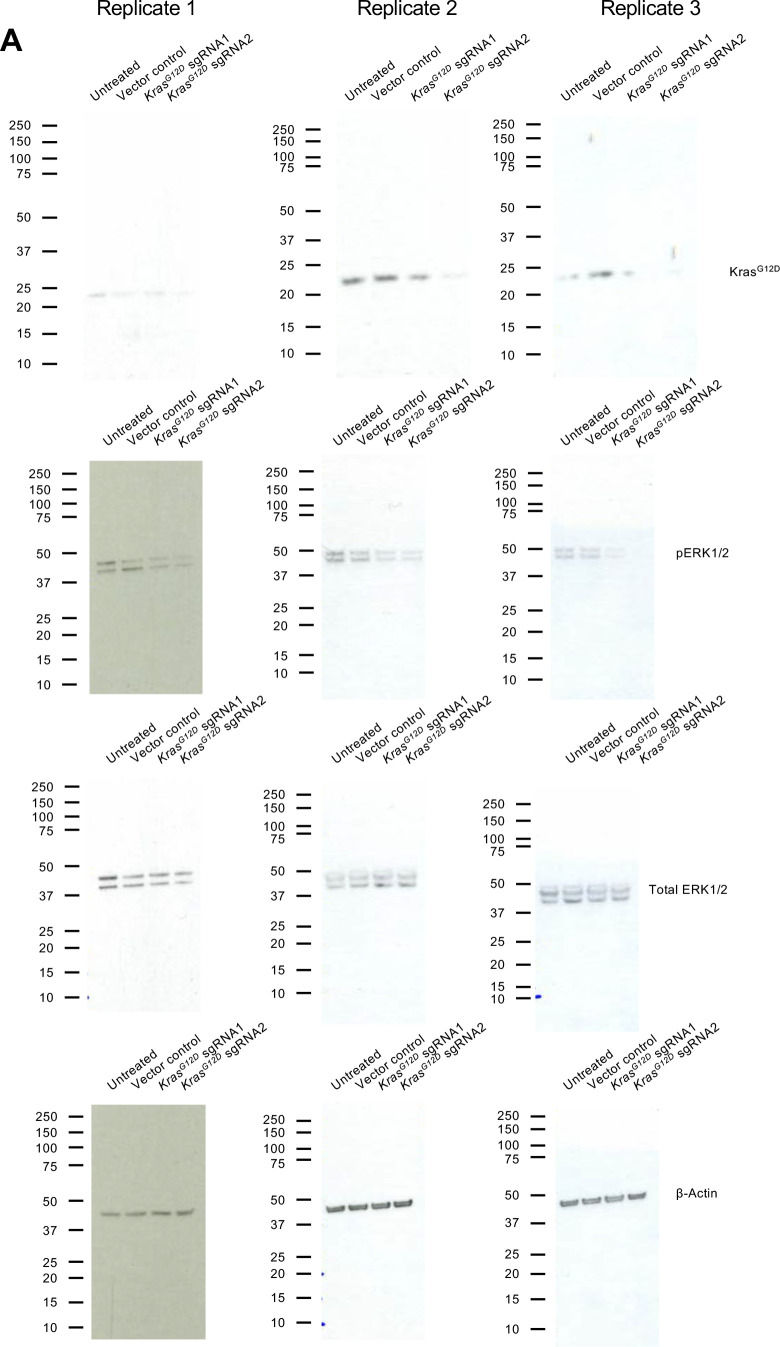
Uncropped Western blots of KPC689 cells treated with exosomes containing plasmid DNA. **(A)** Uncropped full blots of Kras^G12D^, pERK1/2, total ERK, and β-actin. Blots correspond to the images in Fig 2F and quantification in Fig 2F and G.

**Figure S4.**
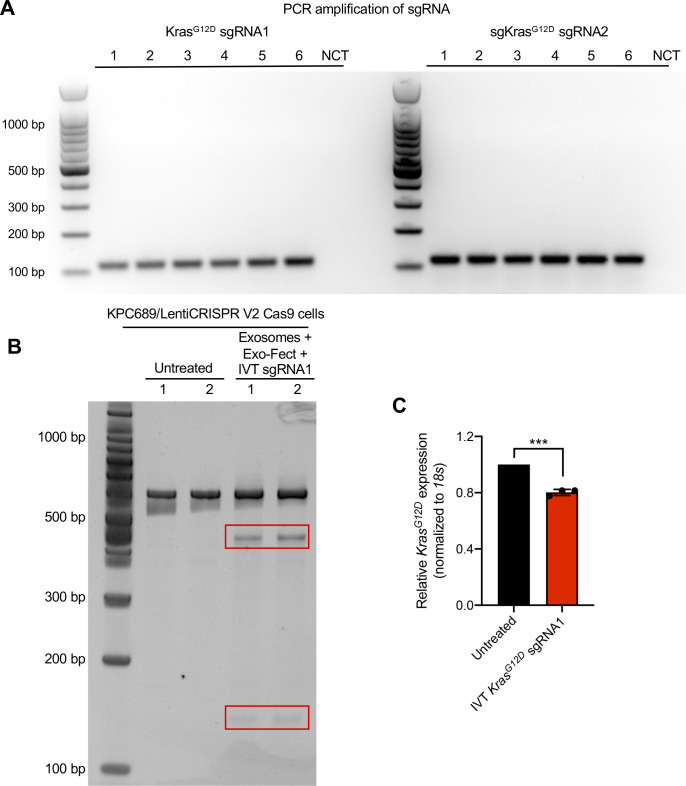
Exosome-mediated delivery of in vitro transcribed (IVT) synthetic guide RNA (sgRNA) induces gene editing in Cas9 overexpressing cells to disrupt oncogenic *Kras*^*G12D*^. **(A)** Generation of IVT sgRNA. Starting from a plasmid sgRNA vector with T7 promoter, *Kras*^*G12D*^ sgRNA1/2 was first PCR-amplified, run on agarose gel to confirm amplicon size, and DNA concentration was quantified with NanoDrop. The PCR products were purified and used as templates for the vitro synthesis of sgRNA. **(B)** HEK293T exosomes were loaded with IVT-*Kras*^*G12D*^ sgRNA1 using Exo-Fect and used to treat Cas9-overexpressing KPC689 cells. Gene editing was tested with T7/Surveyor assay. **(C)** mRNA expression level of *Kras*^*G12D*^ in Cas9-overexpressing cells was assessed by Quantitative PCR. Data are normalized to *18s* and untreated control and presented as mean ± standard deviation. Unpaired *t* test was used to evaluate mean differences based on ΔC_T_ values. ***P < 0.001. Source data are available for this figure.

To evaluate the efficacy of exosomes loaded with CRISPR/Cas9, KPC689 cells were subsequently treated for three consecutive days with 10^9^ HEK293T exosomes (2,500 exosomes/cell) containing 10 μg of plasmid DNA of either (i) LentiCRISPR V2 vector control, or LentiCRISPR V2 plasmid containing (ii) *Kras*^*G12D*^ sgRNA1 or (iii) *Kras*^*G12D*^ sgRNA2. After 3 d, de novo expression of Cas9 mRNA in KPC689 cells was detected across all three exosome treatment groups (Fig 2C). Variations in the level of *cas9* were observed across treatment groups, which may be attributed to variations in plasmid loading into exosomes as the precise mechanism of how Exo-Fect packages DNA into exosomes is not fully understood. In accordance with these data, qPCR showed a significant suppression of *Kras*^*G12D*^ at the transcript level for both cells treated with exosome containing *Kras*^*G12D*^ sgRNA1 and *Kras*^*G12D*^ sgRNA2 relative to exosomes containing the vector control (Fig 2D). Moreover, expression of the WT *Kras* allele was largely unaffected by treatment with exosome containing *Kras*^*G12D*^ sgRNA1 and *Kras*^*G12D*^ sgRNA2, suggesting specific targeting of the mutant allele (Fig 2E). Knockdown of mutant Kras signaling at the protein level was confirmed via Western blot for Kras^G12D^ (Figs 2F and S3A) and its downstream effector pERK1/2 (Figs 2F and G and S3A). The T7/Surveyor assay showed evidence of successful gene editing for KPC689 cells treated with exosomes encapsulating *Kras*^*G12D*^ sgRNA1 and *Kras*^*G12D*^ sgRNA2 but not after treatment with exosomes containing the vector control or in the untreated cells (Fig 2H). To investigate if gene editing is also achieved with in vitro transcribed (IVT) sgRNA in Cas9-overexpressing cells, we PCR-amplified a plasmid with a T7 promoter upstream of the *Kras*^*G12D*^ sgRNA1 sequence and used the PCR product as template for the in vitro synthesis of sgRNA (Fig S4A). We treated a Cas9 KPC689-overexpressing cell line (Fig S1E and F) with HEK293T exosomes loaded with purified IVT-*Kras*^*G12D*^ sgRNA1 using Exo-Fect. We next proceeded to treat Cas9-overexpressing KPC689 cells with 10^9^ exosomes containing IVT-*Kras*^*G12D*^ sgRNA1 every day for three consecutive days and performed a T7/Surveyor assay along with a qPCR to confirm gene editing in the recipient cells. Notably, gene editing and knockdown of *Kras*^*G12D*^ transcript could be observed when IVT sgRNA was loaded into exosomes and delivered into a stable Cas9 overexpressing KPC689 cell line (Fig S4B and C).

Oncogenic mutant *Kras*^*G12D*^ leads to constitutive Ras signaling and enhanced proliferation (18). We conducted an MTT assay to test if exosome-mediated knockdown of *Kras*^*G12D*^ alters cellular proliferation rates. Corroborating the known function of oncogenic RAS in promoting cell growth, we noted a significant suppression in proliferation for KPC689 cells treated with exosomes containing *Kras*^*G12D*^ sgRNA1 and *Kras*^*G12D*^ sgRNA2 plasmid DNA compared to vector control (Fig 2I). Collectively, these results suggest that exosomes are effective nanocarriers for CRISPR by encapsulating and delivering CRISPR/Cas9 encoded plasmid DNA to pancreatic cancer cells to achieve targeted CRISPR-mediated disruption of oncogenic mutant *Kras*^*G12D*^ and suppress proliferation in vitro.

### Exosome-mediated delivery of CRISPR/Cas9 plasmid DNA attenuates tumor progression in a pancreatic cancer mouse model

**Figure S5.**
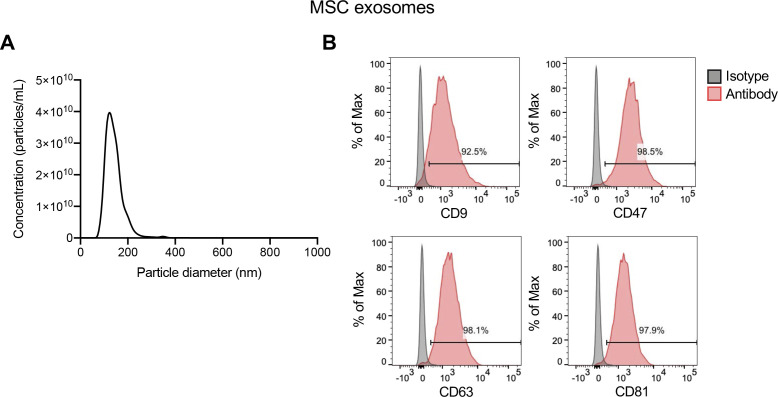
Characterization of mesenchymal stem cell (MSC) exosomes. **(A)** Representative nanoparticle tracking analysis plot of MSC exosomes. **(B)** Representative histogram of flow cytometry analyses of exosomal surface markers (CD9, CD47, CD63, and CD81, indicated in red; isotype control indicated in gray) on MSC exosomes bound to beads. Numbers represent the percentage of positive beads. Source data are available for this figure.

In a proof-of-concept study, the in vivo therapeutic efficacy of CRISPR/Cas9 loaded exosomes was evaluated in a subcutaneous tumor model by implanting cultured KPC689 cells in the dorsum of B6-albino mice. For our in vivo studies we used exosomes purified from bone marrow-derived mesenchymal stem cells (MSCs). Previous studies have demonstrated that MSC exosomes do not elicit signs of toxicity or adverse immune reactions when repeatedly administered in immunocompetent mice every 48 h over the course of 4 mo, as determined by a battery of assays including immunotyping of tissues, histopathological analysis, and cytokine production (12). Moreover, administration of MSC exosomes was not associated with alterations in pancreatic tumor growth (12). NTA and flow cytometry were used to evaluate MSC exosomes for the characteristic size distribution of exosomes and presence of common exosomal surface markers, including the tetraspanins CD9, CD81, and CD63, as well as CD47 (Fig S5A and B), the latter having been linked to enhanced biological activity in vivo and reduced phagocytic clearance during systemic administration (13), and as an abundant protein in exosomes from distinct origins (19). A total of 40 B6-albino mice were allocated into five treatment groups (n = 8 mice in each group) and injected i.v. and intratumorally (IT) every 2 d for 2 wk. One group received 10^9^ MSC exosomes with ExoFect (Exosomes + Exo-Fect), the second group received a plasmid encoding Cas9/*Kras*^*G12D*^ sgRNA1 (*Kras*^*G12D*^ sgRNA1), the third group received a plasmid encoding Cas9/*Kras*^*G12D*^ sgRNA1 with ExoFect (Exo-Fect + *Kras*^*G12D*^ sgRNA1), fourth group received 10^9^ MSC exosomes loaded with a Cas9 vector control using ExoFect (Exosomes + Exo-Fect + vector control), whereas the fifth group received 10^9^ MSC exosomes loaded with a Cas9/*Kras*^*G12D*^ sgRNA1 plasmid using Exo-Fect (Exosomes + Exo-Fect + *Kras*^*G12D*^ sgRNA1). At the end of the treatment period (15 d), tumor volume, tumor weight, tumor burden, and overall body weight were recorded.

**Figure 3.**
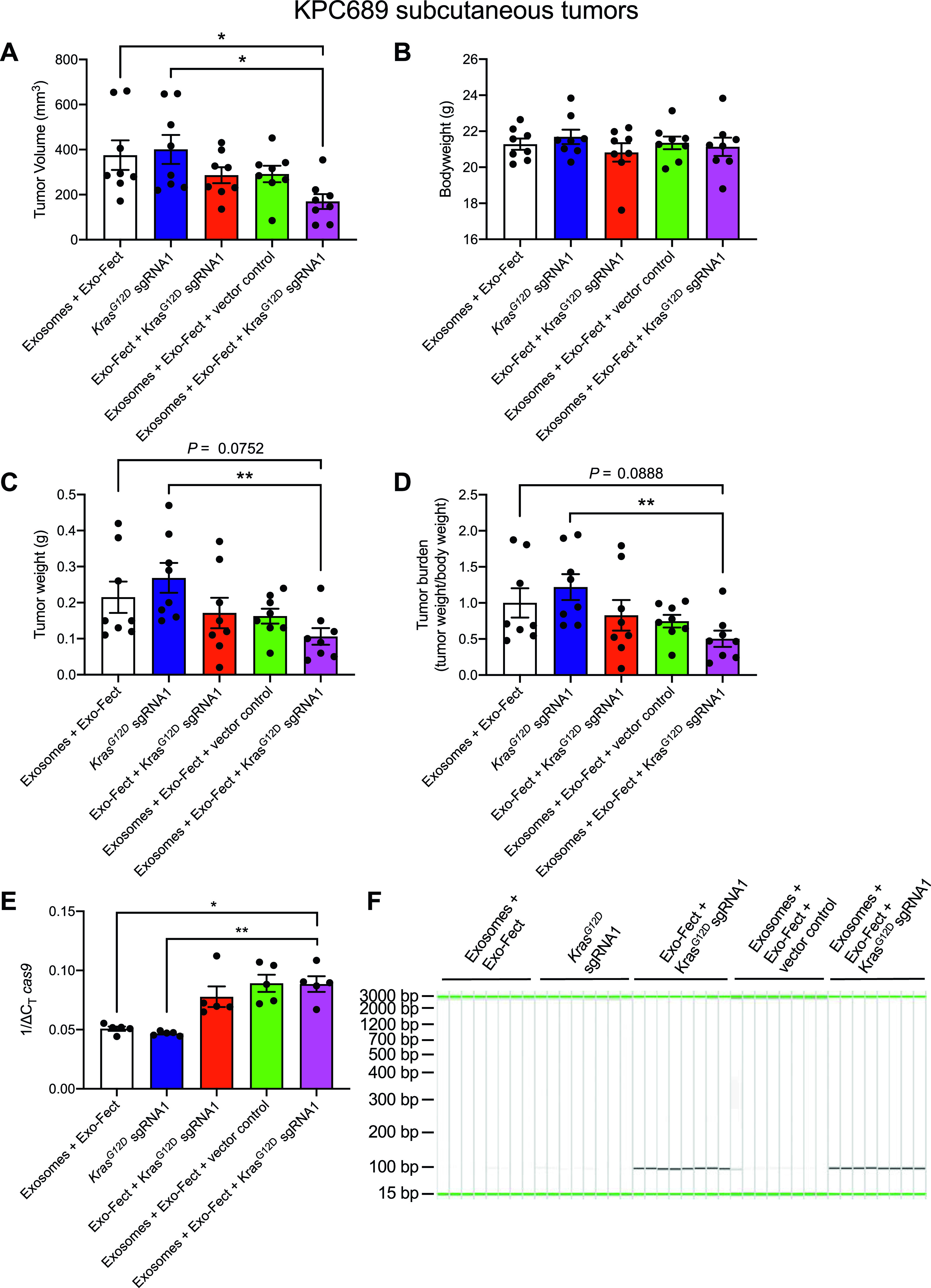
Exosome-mediated delivery of CRISPR/Cas9 inhibits tumor growth in a syngeneic allograft model of pancreatic cancer. KPC689 cells (10^6^) were implanted subcutaneously into the flank of B6-albino mice. The mice were divided into five treatment groups (n = 8 mice in each group) and injected i.v. and intratumorally (I.T.) every other day for 2 wk. **(A)** Tumor volume measurements at end point. **(B)** Body weight of mice at end point. **(C)** Tumor weight at experimental end point. **(D)** Tumor burden (tumor weight/body weight) at end point. **(E)** Expression mRNA levels of intratumoral *cas9* assessed by quantitative PCR (normalized to *18s*). Statistical analysis was performed based on based on ΔC_T_ values. n = 5 mice per group. **(F)** Intratumoral synthetic guide RNA assessed by QIAxcel capillary gel electrophoresis. All measurements are expressed as mean ± SEM. Kruskall–Wallis with Dunn’s multiple comparison test performed. *P < 0.05. Source data are available for this figure.

**Figure S6.**
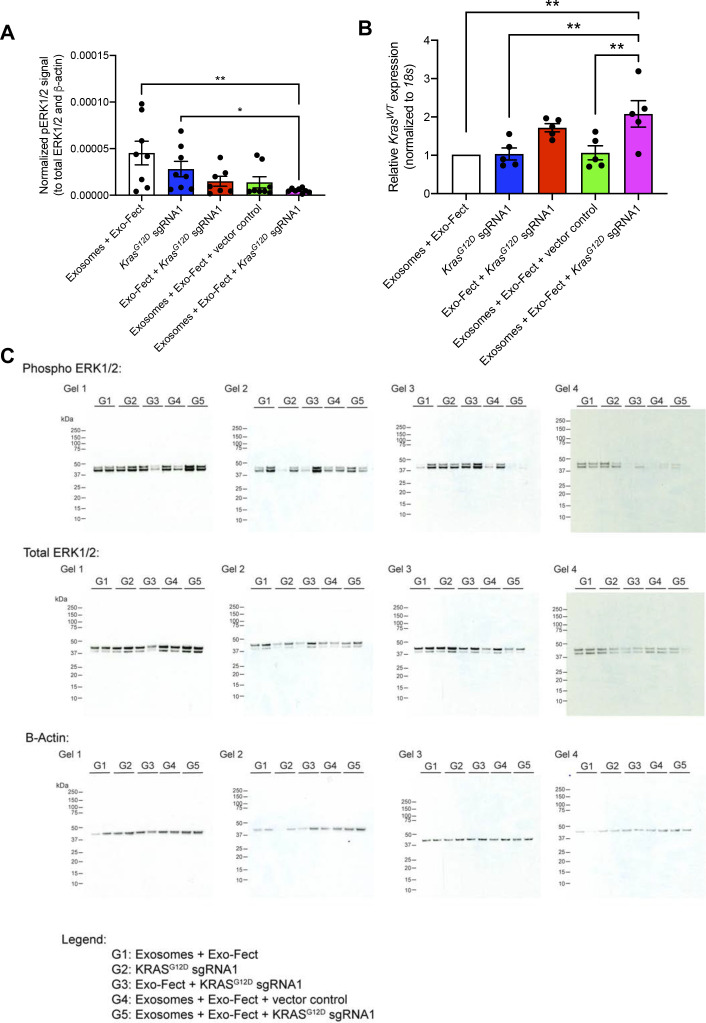
Phospho-ERK activation and WT Kras mRNA levels in syngeneic allograft tumor model. **(A)** Quantification of phospho-ERK1/2 (normalized to total ERK1/2 and β-actin) as assessed via Western blot. Exo-Fect + *Kras*^*G12D*^ synthetic guide RNA1, n = 7 mice; n = 8 mice for all other groups. Data are presented as mean ± SEM. Kruskall–Wallis with Dunn’s multiple comparison test performed. **(B)** Quantitative PCR for WT *Kras* mRNA. Data are normalized to *18s* and Exosomes + Exo-Fect control and presented as mean ± SEM. n = 5 mice per group. One-way ANOVA with Tukey’s multiple comparison test performed. **(C)** Full-length blots for phospho-ERK1/2, total ERK1/2, and β-actin. *P < 0.05, **P < 0.01. Source data are available for this figure.

The treatment group that received MSC exosomes loaded with plasmid encoding for Cas9/*Kras*^*G12D*^ sgRNA1 had the smallest tumor volume at end point relative to other treatment groups (Fig 3A). There was no significant difference in total body weight among the different treatment groups (Fig 3B). Tumor weight as well as tumor burden was also the lowest for the group that received MSC exosomes loaded with the plasmid Cas9/*Kras*^*G12D*^ sgRNA1 (Fig 3C and D). Moreover, the activation of ERK signaling (evaluated via measuring levels of phospho-ERK1/2), a downstream effector of oncogenic KRAS, was reduced in tumors in the treatment group that received MSC exosomes loaded with the plasmid Cas9/*Kras*^*G12D*^ sgRNA1 (Fig S6A and C). There was an increase in the wild-type *Kras* mRNA levels in tumors treated with MSC exosomes loaded with the plasmid Cas9/*Kras*^*G12D*^ sgRNA1 (Fig S6B), which may be attributed to a reduction in tumor cells harboring mutant *Kras* and an increase in non-tumorigenic cells expressing wild-type *Kras*. Cas9 was detectable at the mRNA level in the subcutaneous tumors from the group that received exosomes + Exo-Fect + *Kras*^*G12D*^ sgRNA1 (*P* < 0.05), Exo-Fect + *Kras*^*G12D*^ sgRNA1, and exosomes + Exo-Fect + vector control relative to the group that received exosomes + Exo-Fect alone (Fig 3E). Presence of intratumoral sgRNA was also detectable in the groups that received Exo-Fect + *Kras*^*G12D*^ sgRNA1 and exosomes + Exo-Fect + *Kras*^*G12D*^ sgRNA1 (Fig 3F). Expression of *cas9* in tumors treated with Exo-Fect with *Kras*^*G12D*^ sgRNA was observed, indicating that the transfection reagent can deliver plasmid DNA without exosomes; however, mice treated with exosomes containing *Kras*^*G12D*^ sgRNA had further reduced tumor burden, suggesting that exosome-based delivery of DNA may have improved therapeutic efficacy. Taken together, CRISPR/Cas9 plasmid-loaded exosomes administered intravenously and intratumorally, can effectively target oncogenic KRAS^G12D^ and suppress tumor growth in vivo in the context of a syngeneic subcutaneous tumor model.

We next used an orthotopic tumor model of pancreatic cancer to further assess the in vivo efficacy of exosome-mediated delivery of CRISPR/Cas9 via i.p. administration. In short, the growth of luciferase-expressing KPC689-GFP-Luc^+^ cells orthotopically injected in the pancreas was monitored via bioluminescent imaging. 3 d after orthotopic tumor implantation, the mice were divided into four groups (n = 3 mice in each treatment group) and injected i.p. every other day for 3 wk with bioluminescent imaging performed at regular intervals on day 0 (baseline), day 10, day 20, and day 24 (end point). The first group received Exosome + Exo-Fect, the second group received *Kras*^*G12D*^ sgRNA1 in its free form, a third group received Exosomes + Exo-Fect + vector control and the last group Exosomes + Exo-Fect + *Kras*^*G12D*^ sgRNA1. At end point, tumor expression levels of *cas9*, sgRNA and *Kras*^*G12D*^ mRNA were measured as a proxy for gene editing.

**Figure 4.**
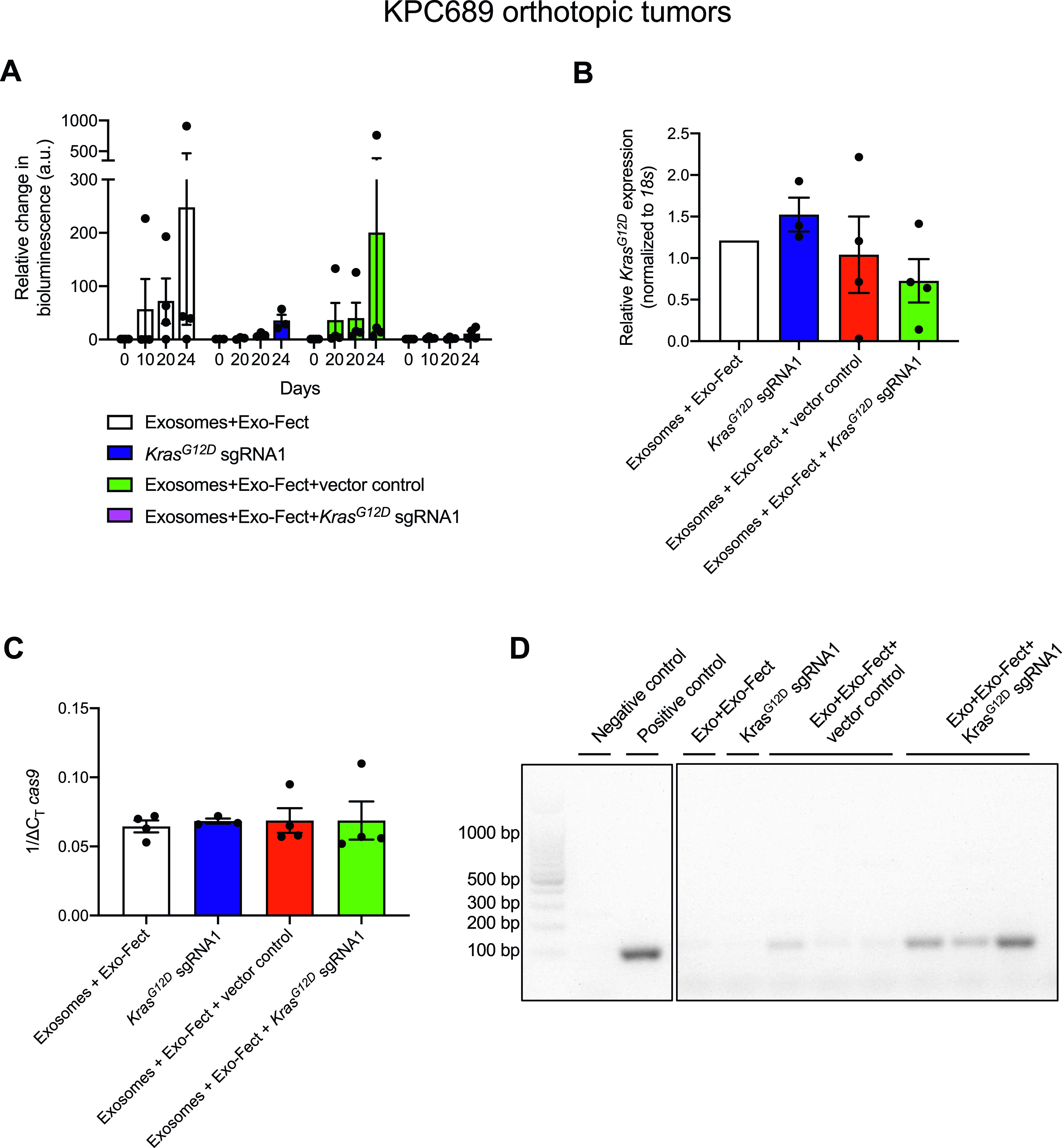
Exosome-mediated delivery of CRISPR/Cas9 reduces tumor growth in an orthotopic model of pancreatic cancer. KPC689-GFP-Luc^+^ cells (5 × 10^5^) were orthotopically injected into the pancreas of B6-albino mice at day 0 (D0). The mice were enrolled 3 d post orthotopic tumor implantation, divided into four treatment groups (n = 3–4 mice in each group) and injected i.p. every other day for 3 wk. **(A)** Tumor growth was tracked with bioluminescent imaging at day 0 (D0), day 11 (D11), day 20 (D20), and day 24 (D24). The bioluminescence was normalized to the photon flux observed D0 and the relative change in bioluminescence is reported. Two-way ANOVA was performed with Tukey’s multiple comparison test to compare differences in the mean at different days/time points among the different groups. **(B)** Tumor mRNA expression levels of *Kras*^*G12D*^ at end point was evaluated with Quantitative PCR. Data are normalized to *18s* levels and the Exosomes + Exo-Fect control group. One-way ANOVA with Tukey’s multiple comparisons test was used to evaluate mean differences among groups based on ΔC_T_ values. **(C)** Tumor mRNA expression levels of *cas9* was evaluated with Quantitative PCR. Data are normalized to *18s* levels. One-way ANOVA with Tukey’s multiple comparisons test was used to evaluate mean differences among groups based on ΔC_T_ values. **(D)** Tumor expression level of synthetic guide RNA (∼100 bp) was also confirmed by agarose gel electrophoresis of reverse transcribed cDNA. The data are expressed as mean ± SEM. Source data are available for this figure.

Tumor growth was observed in all treatment groups. The treatment group that received MSC exosomes loaded with Cas9/*Kras*^*G12D*^ sgRNA1 plasmid showed the smallest relative increase in tumor size from baseline to end point compared with all other groups as assessed by bioluminescent imaging, although statistical significance was not reached, likely due to the small sample size (Fig 4A). Corroborating the trend of suppressed tumor growth seen with bioluminescent imaging, we also noted a trend for reduced levels of *Kras*^*G12D*^ mRNA in the group treated with exosomes containing Cas9/*Kras*^*G12D*^ sgRNA1 plasmid relative to all other groups, which however did not reach statistical significance (Fig 4B). At end point, qPCR confirmed a trend for higher levels of de novo expression of intratumoral *cas9* mRNA in groups that received treatment with exosomes containing Cas9 plasmid as empty vector or Cas9/*Kras*^*G12D*^ sgRNA1 plasmid, but the difference was not statistically significant compared with the control group Exosomes + Exo-Fect (Fig 4C). The presence of intratumoral sgRNA was not observed for Cas9/*Kras*^*G12D*^ sgRNA1 injected on its own, highlighting that plasmid alone without encapsulation in exosomes cannot effectively reach tumor cells in vivo for successful gene editing, possibly owing to enzymatic degradation by nucleases (Fig 4D). These data confirmed that exosomes can deliver CRISPR/Cas9 plasmid DNA; however, further delivery optimization and validation is necessary to evaluate if exosomes are effective carriers of CRISPR/Cas9 to orthotopic pancreatic tumors.

## Discussion

*KRAS* is the most frequently mutated oncogene (95%) in pancreatic adenocarcinoma with a causal role in cancer initiation, propagation and maintenance (20). Despite decades of intensive efforts, the development of effective small molecule therapeutics or antibodies for inhibiting activating *KRAS* mutations has remained largely elusive, necessitating alternative approaches (21). Here we report a CRISPR/Cas9-based strategy aimed at knocking-out the *Kras*^*G12D*^ oncogenic mutation in vitro and in vivo using exosomes as a nonviral delivery platform. Unlike previously used RNAi-based strategies that selectively inhibit mutant KRAS mRNA post-transcriptionally, CRISPR/Cas9 offers the theoretical advantage of complete knockout of the mutant allele from its endogenous genomic locus, rather than knockdown of transcript expression, while obviating the need for continuous delivery to maintain target mRNA/protein suppression.

Our results provide proof-of-concept on the feasibility of non-autologous exosomes to act as nanocarriers for encapsulating and delivering CRISPR/Cas9 plasmid DNA to inhibit mutant *Kras*-dependent pancreatic cancer cell proliferation in vitro and tumor growth in vivo. Caution is advised, however, in light of evidence that *KRAS* inhibition using CRISPR/Cas9–mediated gene editing of oncogenic *KRAS* may be dispensable for a subset of pancreatic cancer cell clones that are able to survive without it (22). Corroborating to this point, other lines of evidence also suggest that while inducible extinction of *Kras* in genetically engineered mouse models results in complete regression of pancreatic tumors in the short term, a subset of those tumors recur in the long term by escaping *Kras* oncogenic addiction through a YAP1-mediated transcriptional program (23). While promising, our results need to be framed in the broader context of the significant challenges that remain before CRISPR/Cas9 technology becomes a realistic technology for use in cancer therapy. Regarding the on-target activity, it should be noted that the exosome-mediated delivery of CRISPR/Cas9 imparted only a moderate knockdown (∼58% suppression) on the target *Kras*^*G12D*^ transcript levels even in the in vitro context (Fig 2D). This is not surprising because of the sheer number of barriers that need to be overcome for the exosome uptake and delivery of the CRISPR/Cas9 cargo to the nucleus for editing the genome. In an in vitro setting the rate of editing efficiency is largely determined by the various delivery barriers encountered in the journey from the cell surface to nuclear entry, starting from the rate of endocytosis at the level of the cell membrane, to the intracellular transport kinetics, endosomal release, and nuclear transport. Once in the nucleus, factors such as the relative abundance of the individual components of the gene-editing machinery and the accessibility of the target locus in the chromosome become important as well. Small molecule drugs that increase the rate of endosomal escape of exosomal cargo into the cytosol by manipulating the acidification and/or lipid composition of maturing endosomes may potentially increase the rate of gene editing in an in vitro setting (24). Systemic in vivo delivery of the exosomal CRISPR cargo presents additional transport barriers including extravasation from the blood vessel endothelium and migration through the tissue-specific interstitial space before it reaches the target cells. It is important to acknowledge however that the threshold for gene editing in the target cells may not be particularly high for a therapeutic outcome to be manifested. This likely depends on factors extending beyond delivery efficiency, such as the relative fitness of edited cells versus non-edited cells and whether the gene of interest operates in a cell-autonomous versus non-cell autonomous fashion (25). Despite the modest suppression in Kras^G12D^ mRNA and protein with exosome-mediated delivery of CRISPR/Cas9, we noticed a reduction in proliferation of tumor cells in vitro and tumor growth inhibition in vivo at a similar level. Additional studies focused on developing methods to improve CRISPR/Cas9 encapsulation in exosomes, gene editing efficiency in recipient cells, and the delivery of exosomes to tumors will be of value to fully realize the therapeutic potential of exosome-based delivery vehicles.

The targeting specificity of our CRISPR approach relies on two factors— the sgRNA design itself and exosome-related organ-specific tropism. First, the sgRNA is designed to selectively target the mutant *Kras* allele (G12D: GGT > GAT in codon 12 of exon 2) in cancer cells while sparing, at least theoretically, nonmalignant cells harboring the wild-type allele. Second, exosomes administered systemically via i.p. have shown organ-specific tropism and are known to preferentially accumulate within a few key organs including the liver, lung, and pancreas (13). In principle, exosomes can also be functionalized with targeting moieties to promote selective cancer cell targeting or be exploited for organ-specific tropism and/or preferential passive accumulation to tumor sites via the EPR effect (26) to enhance their efficacy. CRISPR/Cas9 loaded cancer-derived exosomes for instance have been shown to preferentially accumulate in ovarian cancer tumors presumably because of their cell tropism (14). Further insight into the mechanisms regulating exosome entry may provide additional routes to improve delivery specifically to cancer cells.

The CRISPR system is of bacterial origin and adaptive immune responses can be directed against Cas9 or components of the delivery system. Several studies have reported that humans harbor preexisting humoral and cell-mediated adaptive immunity against the bacterial-derived Cas9 evident by the presence of anti-Cas9 antibodies and Cas9-specific cellular responses potentially because of exposure via the microbiome or preexisting infection with *Streptococcus pyogenes* (10, 27, 28, 29). This pre-existing immunity has important implications as it may neutralize the effectiveness of gene editing or potentially cause serious safety issues. Exosome-mediated delivery of the CRISPR gene editing elements largely bypasses immunogenicity concerns as it provides an immune-privileged protective delivery vehicle (unlike viral vectors or PEGylated synthetic nanoparticles) but cannot necessarily mitigate unwanted off-target effects which are partly intrinsic to the CRISPR machinery. The possibility of off-target effects can vary depending on the cargo format and the nature of the intended gene correction. In terms of cargo type our exosome-loading approach entailed the use of plasmid DNA (low-cost and easy-to-use) that induces long-term constitutive expression of the bacterial Cas9 protein, and as such we cannot rule out the possibility of Cas9-induced mutagenesis at the wild-type allele, which could potentially result in, albeit at low efficiency, generation of unintended oncogenic alleles. Moreover, risk of off-target effects can be theoretically increased when targeting point mutations (G12D) via the NHEJ-mediated DNA repair mechanism as the difference in a single nucleotide may not be sufficiently different from the functioning allele on the homologous chromosome to be distinguished by the *Sp.Cas9* nuclease, potentially leading to an undesirable complete loss of protein function.

There is no clear-cut way to completely eliminate the possibility of unintended off-target effects with CRISPR regardless of the delivery method used, as it is a function of both the intrinsic properties of the gene editing machinery, namely the specific Cas9 enzyme and gRNA of choice, as well as the cargo format (plasmid, mRNA, or RNP) and the delivery vehicle. A sound rationale for mitigating off-target activity to the highest degree possible would be a combinatorial approach where exosomes are loaded with a more transiently active CRISPR cargo format in the form of pre-formed RNPs, ideally making use of truncated sgRNAs (tru-gRNAs) and an optimized high-fidelity Cas9 variant (30). Some lines of evidence suggest that the highest on-target efficiency and lowest frequency of off-target events is achieved with delivery of RNPs or in cells stably expressing Cas9 treated with IVT sgRNA (31). However, these systems require delivery of both Cas9 and sgRNA into the same cell, which remains a challenge in vivo where delivery and editing efficiency remain low. Trade-offs between maximizing on-target efficiency and minimizing off-target events need to be accounted for in addition to considerations regarding overall complexity, ease of large-scale production, cost and cargo stability.

In conclusion, engineered exosomes can serve as a delivery platform for CRISPR/Cas9 DNA to inhibit oncogenic *Kras*^*G12D*^ in vitro and suppress tumor growth in vivo.

## Materials and Methods

### CRISPR plasmids and sgRNA sequences

The sequences of sgRNAs targeting the mouse *Kras*^*G12D*^ gene are listed in Table 1. Oligos were synthesized by Sigma-Aldrich. The synthesized paired oligos were diluted in sterile water and annealed in a thermal cycler. The annealed oligos were then cloned into the lentiCRISPR v2 sgRNA backbone after *BsmBI* digestion or pSpCas9(BB)-2A-GFP (PX458) sgRNA backbone after *BbsI* digestion. lentiCRISPR v2 (plasmid #52961; http://n2t.net/addgene:52961; RRID: Addgene_52961; Addgene) and pSpCas9(BB)-2A-GFP (PX458) (plasmid #48138; http://n2t.net/addgene:48138; RRID: Addgene_48138; Addgene) were purchased from Addgene.

**Table 1.** Sequences of synthetic guide RNA (sgRNA) targeting *Kras*^*G12D*^.

sgRNA	sgRNA sequence (5′ to 3′)
sgRNA1 *Kras*^*G12D*^ forward	CACCGGTGGTTGGAGCTGATGGCGT
sgRNA1 *Kras*^*G12D*^ reverse	AAACACGCCATCAGCTCCAACCACC
sgRNA2 *Kras*^*G12D*^ forward	CACCGCTTGTGGTGGTTGGAGCTGA
sgRNA2 *Kras*^*G12D*^ reverse	AAACTCAGCTCCAACCACCACAAGC

### Cell culture

KPC689 cells were cultured in RPMI (Corning) supplemented with 10% FBS (Gemini) and 1% penicillin-streptomycin (Corning). The KPC689 cancer cell line was isolated from an autochthonous pancreatic tumor of *Pdx1*^cre/+^; *LSL-Kras*^*G12D/+*^*; LSL-Trp53*^*R172H/+*^ (KPC) mice as previously described (17). Bone marrow-derived MSCs were obtained from the Cell Therapy Laboratory at the University of Texas MD Anderson Cancer Center and cultured in αMEM (Corning) supplemented with 20% FBS, 1% nonessential amino acids (Corning), 1% L-glutamine (Corning), and 1% penicillin-streptomycin. HEK293T/17 (293T) cells were cultured in DMEM (Corning) supplemented with 10% FBS and 1% penicillin–streptomycin. HEK293T cells were obtained from ATCC and short tandem repeats (STR) fingerprinting performed to confirm their identity. The cells were screened and tested negative for mycoplasma. All cells were cultured in 37°C and 5% CO_2_.

### Isolation and purification of exosomes

For cell culture supernatant derived exosomes, the cell lines were cultured until 80% confluence, washed twice with PBS, and 35 ml of serum-free media was added to the cells. For MSC exosome isolation, serum-free media consisted of αMEM (Corning) supplemented with 1% nonessential amino acids (Corning), 1% L-glutamine (Corning), and 1% penicillin–streptomycin. Supernatant was collected from cells that were cultured in the conditioned medium for 48 h, and was centrifuged at 800*g* for 5 min, and 2,000*g* for 10 min. This resulting supernatant was then filtered using 0.2 μm filters (Corning). Exosomes were pelleted by ultracentrifugation (Beckman) at 100,000*g* in an SW32 Ti rotor for 3 h. The supernatant was aspirated and the pellet was resuspended in PBS. The size and concentration of exosomes was verified using NTA (Malvern NanoSight LM10) and manufacturer’s software.

### DNA/RNA extraction

DNA and RNA from cultured cells were extracted using AllPrep DNA/RNA Mini kit (QIAGEN) according to the manufacturer’s instructions. DNA and RNA concentration were quantified using NanoDrop 2000 spectrophotometers (Thermo Fisher Scientific). DNA was extracted from exosomes using QIAamp DNA Micro kit (QIAGEN) according to the manufacturer’s instructions. DNA concentration was quantified using Qubit dsDNA high-sensitivity assay according to manufacturer’s instructions (Thermo Fisher Scientific). For DNase treatment, exosome and plasmid samples were treated with 200 U/ml DNase (Promega) according to the manufacturer’s instructions before DNA isolation.

### Quantitative real-time PCR analyses

cDNA was synthetized using High Capacity cDNA Reverse Transcription Kit (Applied Biosystems). qPCR analyses were performed on an Applied Biosystems Quantstudio 7 using SYBR Green Master Mix (Applied Biosystems). The transcripts of interest were normalized to *ACTB* or *18s* transcript levels. Primer sequences are shown in Table 2. The data are presented as relative fold change or 1/ΔCt. Each reaction included three technical replicates, which were averaged to define one biological replicate. Statistical analyses were performed on ΔCt of biological replicates (mice or independent experiments) and the results expressed as relative fold change.

**Table 2.** Quantitative PCR primer sequences.

Gene	Sequence
*cas9* forward	5′-GCCAGATCCTGAAAGAACAC-3′
*cas9* reverse	5′-TCCTGGTCCACGTACATATC-3′
*ACTB* forward	5′-CATGTACGTTGCTATCCAGGC-3′
*ACTB* reverse	5′-CTCCTTAATGTCACGCACGAT-3′
*Kras*^*G12D*^ forward	5′-ACTTGTGGTGGTTGGAGCAGA-3′
*Kras*^*G12D*^ reverse	5′-TAGGGTCATACTCATCCACAA-3′
WT *Kras* forward	5′-CAAGAGCGCCTTGACGATACA-3′
WT *Kras* reverse	5′-CCAAGAGACAGGTTTCTCCATC-3′
*18s* forward	5′-GTAACCCGTTGAACCCCATT-3′
*18s* reverse	5′-CCATCCAATCGGTAGTAGCG-3′

### T7/surveyor assay

Genomic DNA was extracted for confirmation of the indels or mutations. Each sgRNA genomic target site was prepared using a PCR amplicon with specific primers as shown in Table 3. PCR amplicons were purified, and 250 ng was reannealed using a thermocycler and then digested with T7 endonuclease Ι (T7E1; New England Biolabs) according to the manufacturer’s instructions. Digested DNA was analyzed using polyacrylamide gel.

**Table 3.** T7E1 surveyor assay primer sequences.

Primer	Sequence
*Kras*^*G12D*^ synthetic guide RNA1 forward	5′-GTGTGTCCACAGGGTATAGCG -3′
*Kras*^*G12D*^ synthetic guide RNA1 reverse	5′-TCTTTTTCAAAGCGGCTGGC -3′

### Loading of exosomes with plasmid DNA

Cargo loading in exosomes was achieved using Exo-Fect Exosome Transfection Kit (System Biosciences) according to manufacturer’s instructions. Direct transfection of HEK293T cells was also used for loading of plasmid DNA into exosomes. HEK293T cells were transfected with lenti-Cas9 vectors using Lipofectamine 2000 for 72 h and then selected with 1 μg/ml puromycin for 10 d to obtain stable HEK293T/lenti-Cas9 cells. The stables cells were cultured with 1 μg/ml puromycin containing selection medium. The cell medium was replaced with fresh medium without FBS. After 48 h culture, the exosomes were isolated from the harvested cell medium with the same purification reagents mentioned above.

### Western blotting

To deduce the protein levels in cell, exosome, or KPC689 subcutaneous tumor lysates, cells or exosomes were homogenized in urea lysis buffer (8 M Urea, 2.5% SDS) with protease inhibitors added (cOmplete protease inhibitor cocktail; Sigma-Aldrich). For assessment of ERK1/2 activation, samples were lysed as described above, with the addition of phosphatase inhibitor cocktail PhosSTOP (Roche). Protein lysates were normalized using bicinchoninic acid (BCA) protein assay kit (Pierce, Thermo Fisher Scientific) or Qubit Protein assay (Invitrogen). Protein lysates were loaded onto acrylamide gels for electrophoretic separation of proteins under denaturing conditions and transferred onto polyvinylidene fluoride (PVDF) membranes by Trans-Blot Turbo Transfer System (1704150; Bio-Rad). The membranes were then blocked for 2 h at room temperature with 5% non-fat dry milk or 5% BSA, in TBS with 0.1% Tween-20, and incubated overnight at 4°C with primary antibodies. Secondary antibodies were incubated for 2 h at room temperature. The primary and secondary antibodies used are listed in Table 4. Washes after antibody incubations were done with an orbital shaker, five times at 5-min intervals, with TBS containing 0.1% Tween-20. Membranes were developed with chemiluminescent reagents from Pierce, according to the manufacturer’s instructions.

**Table 4.** Antibodies used for Western blot analysis.

Target protein	Host species	Dilution	Vendor, cat. no.
CD81	Mouse	1:1,000	Santa Cruz, sc-166029
Alix	Mouse	1:1,000	CST, 2171
β-actin	Rabbit	1:1,000	CST, 4970
Calnexin	Mouse	1:200	Santa Cruz, sc-23954
Ras^G12D^	Rabbit	1:1,000	CST, 14429
p44/42 MAPK (Erk1/2)	Rabbit	1:1,000	CST, 9102
Phospho-p44/42 MAPK (Erk1/2) (Thr202/Tyr204)	Rabbit	1:1,000	CST, 4376
Cas9	Mouse	1:500	Abcam, ab191468
HRP-conjugated β-actin	-	1:25,000	Sigma-Aldrich, A3854
Anti-rabbit HRP-conjugated	-	1:5,000	CST, 7074
Anti-mouse HRP-conjugated	-	1:1,000	R&D, HAF007

### Flow cytometry analyses of exosomes

MSC exosomes (3 × 10^9^) were diluted in 200 μl of PBS, 10 μl of aldehyde latex beads (Invitrogen) added, and the samples rotated at room temperature for 15 min. Samples were diluted with 600 μl of PBS and incubated overnight at 4°C. 400 μl of 1 M glycine was added and the beads incubated for 30 min at room temperature, followed by centrifugation at 8,000*g* for 1 min. Beads were resuspended in 10% BSA in PBS, blocked for 1 h at room temperature, and centrifuged at 8,000*g* for 1 min. Samples were incubated with 2.5 μg/ml of primary antibody (CD9, SAB4700092; Sigma-Aldrich; CD47, 14-0479; eBioscience; CD63, 556019, BD; CD81, 555675, BD; mouse IgG1κ isotype control, 555746, BD) in 20 μl of 2% BSA in PBS for 1 h at room temperature. Samples were washed three times with 200 μl of 2% BSA in PBS, followed by incubation in 100 μg/ml Alexa Fluor 488 donkey anti-mouse IgG (A21202; Invitrogen) in 20 μl of 2% BSA for 1 h at room temperature. Samples were washed three times with 200 μl of 2% BSA in PBS and analyzed using a BD LSR Fortessa X-20. Data analysis was performed in FlowJo (BD) and positivity determined based on signal in the isotype control.

### Cell viability assay/MTT

Cell viability/proliferation was determined using the standard MTT (3-[4,5-dimethylthiazol-2-yl]-2,5-diphenyltetrazolium bromide) assay. KPC689 cells were seeded at an initial density of ∼5 × 10^4^ cells/well in 48 well plates. The purple formazan crystals were dissolved in DMSO, transferred into a 96-well plate (100 μl/well) and the absorbance was recorded on a microplate reader at an optical density of 490 nm at 24, 48, and 72 h. Wells in triplicate were averaged to define one independent biological experiment and three independent experiments were averaged to define the mean and SEM for each treatment group and time points.

### In vivo experiments

Mice were housed in individually ventilated cages on a 12 h light:12 h dark cycle at 21°C–23°C and 40–60% humidity. Mice were allowed free access to an irradiated diet and sterilized water. Female B6-albino mice (Jackson Laboratory) between 8 and 10 wk of age were used. 1 million KPC689 cells were injected subcutaneously into the flank of mice. After the tumor volume reached ∼100 mm^3^, 10^9^ CRISPR/Cas9-loaded exosomes (10^9^ exosomes, 10 μg plasmid DNA) were administered intravenously and intratumorally every Monday, Wednesday, and Friday for 2 wk. Tumor sizes and body weight were measured three times per week. Mice were euthanized 2 d after the last treatment and tumors were harvested at the end point of the experiments. Tumor volume was calculated as V (mm^3^) = 0.52 × length × width^2^.

For the orthotopic model, 5 × 10^5^ KPC689-GFP-Luc^+^ cells were injected into the tail of the pancreas of B6-albino mice. Tumor growth was monitored by bioluminescent imaging (IVIS 200 small animal imaging system; PerkinElmer). Treatment with exosomes (10^9^ exosomes, 10 μg plasmid DNA) administered i.p. every other day started 3 d post-tumor cell induction and continued for 3 wk. All animal experiments were reviewed and approved by the Institute for Care and Use Committee at UT MD Anderson Cancer Center.

### Statistical analyses

Statistical analyses were performed on the mean values of biological replicates in each group. All results were expressed as mean ± standard deviation or SEM, as indicated in the figure legends. For comparison of two groups, an unpaired *t* test was used. The significance (*P*-value) of the difference among three or more groups was evaluated using one-way ANOVA with post-hoc Tukey test for multiple comparisons. When three or more groups with significantly different standard deviations were compared, Brown–Forsythe ANOVA with Dunnett’s T3 multiple comparisons test was used. For non-normally distributed data, Kruskall–Wallis with Dunn’s multiple comparison test was performed. Two-way ANOVA was used for analysis of multiple groups over time. The statistical tests used are indicated in the figure legend. Values of *P* < 0.05 were considered statistically significant.

## Supplementary Material

Reviewer comments
