# Supplementary figures and images for: Exosome-mediated delivery of CRISPR/Cas9 for targeting of oncogenic KrasG12D in pancreatic cancer
Source: Life Sci Alliance. 2021 Jul 19;4(9):e202000875. doi: 10.26508/lsa.202000875 (PMC8321670; doi:10.26508/lsa.202000875)

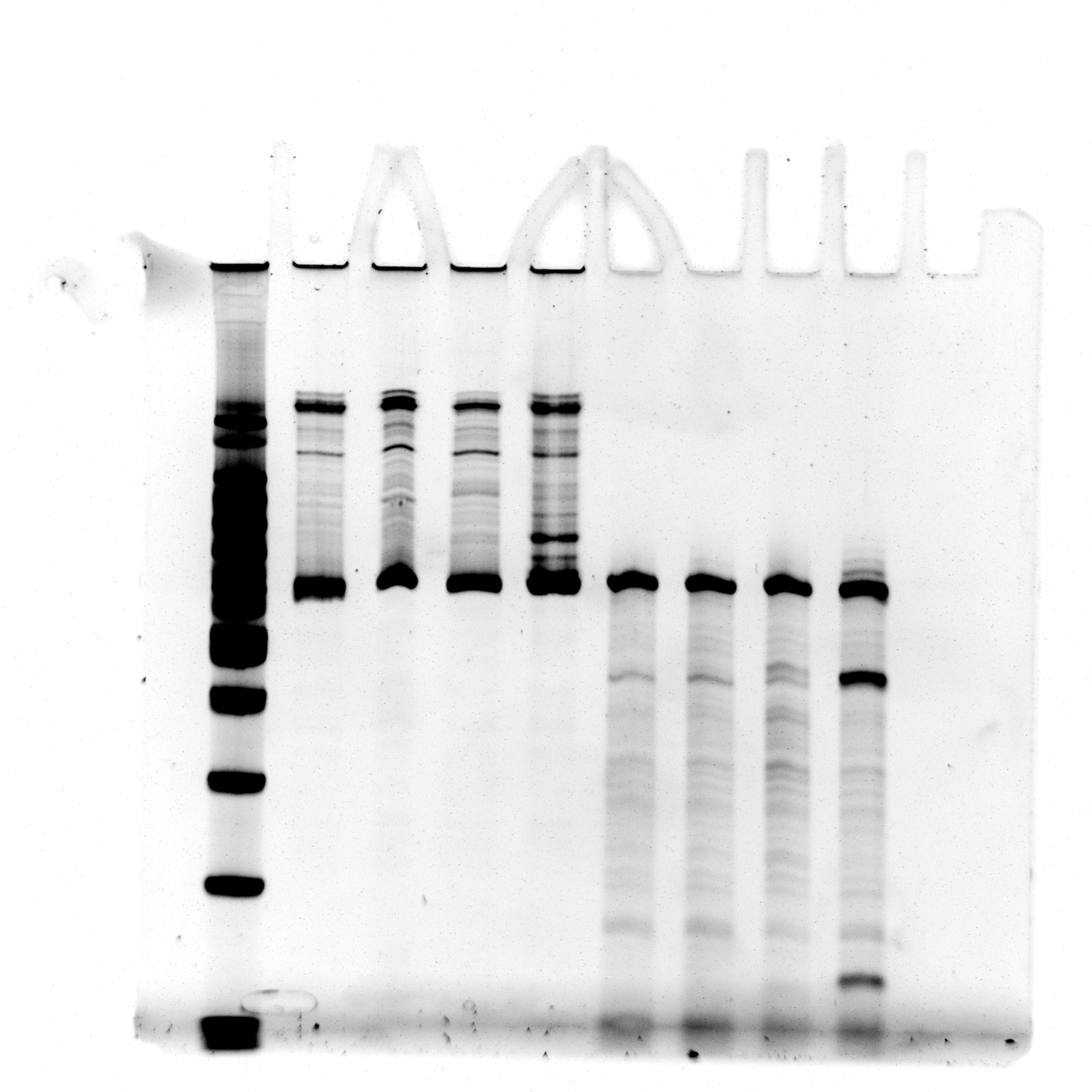

Supplement: Supplementary file 5 [file LSA-2020-00875_SdataF2.2.tif]
