# Supplementary material for: Exosome-mediated delivery of CRISPR/Cas9 for targeting of oncogenic KrasG12D in pancreatic cancer
Source: Life Sci Alliance. 2021 Jul 19;4(9):e202000875. doi: 10.26508/lsa.202000875 (PMC8321670; doi:10.26508/lsa.202000875)
Supplement: Reviewer comments [file LSA-2020-00875_review_history.pdf]

Life Science Alliance

Exosome-mediated delivery of CRISPR/Cas9 for targeting of oncogenic KrasG12D in pancreatic cancer

Kathleen McAndrews, Fei Xiao, Antonios Chronopoulos, Valerie LeBleu, Fernanda Kugeratski and Raghu Kalluri
DOI: https://doi.org/10.26508/lsa.202000875

Corresponding author(s): Raghu Kalluri (University of Texas MD Anderson Cancer Center)

Review Timeline:

Submission Date:	2020-08-13
Editorial Decision:	2020-09-16
Revision Received:	2020-12-15
Editorial Decision:	2021-01-29
Revision Received:	2021-04-11
Editorial Decision:	2021-05-24
Revision Received:	2021-06-25
Editorial Decision:	2021-06-28
Revision Received:	2021-07-02
Accepted:	2021-07-05

Scientific Editor: Eric Sawey, PhD

Transaction Report:

(Note: With the exception of the correction of typographical or spelling errors that could be a source of ambiguity, letters and reports are not edited. The original formatting of letters and referee reports may not be reflected in this compilation.)

No full Peer Review Process File is available with this article, as the authors have chosen not to make the review process public in this case.

September 16, 2020

Re: Life Science Alliance manuscript #LSA-2020-00875

Dr. Raghu Kalluri
University of Texas MD Anderson Cancer Center
Department of Cancer Biology
Beth Israel Deaconess Medical Center Division of Matrix Biology 330 Brookline Ave
Center for Life Sciences 11-087 3 Blackfan Circle
Houston, TX 77030

Dear Dr. Kalluri,

Thank you for submitting your manuscript entitled "Exosome-mediated delivery of CRISPR/Cas9 for targeting of oncogenic KRASG12D in pancreatic cancer" to Life Science Alliance. We apologize for this delay in getting our decision to you.

The manuscript was assessed by expert reviewers, whose comments are appended to this letter.

We would like to invite you to revise your manuscript in accordance to the reviewers' concerns and re-submit to Life Science Alliance.

To upload the revised version of your manuscript, please log in to your account:
https://lsa.msubmit.net/cgi-bin/main.plex
You will be guided to complete the submission of your revised manuscript and to fill in all necessary information.

We would be happy to discuss the individual revision points further with you should this be helpful. While you are revising your manuscript, please also attend to the below editorial points to help expedite the publication of your manuscript. Please direct any editorial questions to the journal office.

The typical timeframe for revisions is three months. Please note that papers are generally considered through only one revision cycle, so strong support from the referees on the revised version is needed for acceptance. When submitting the revision, please include a letter addressing the reviewers' comments point by point.

Thank you for considering Life Science Alliance as an appropriate venue for your research. We look forward to receiving the revised manuscript.

Sincerely,

Shachi Bhatt, Ph.D.
Executive Editor
Life Science Alliance

A. THESE ITEMS ARE REQUIRED FOR REVISIONS

-- A letter addressing the reviewers' comments point by point.

-- An editable version of the final text (.DOC or .DOCX) is needed for copyediting (no PDFs).

-- High-resolution figure, supplementary figure and video files uploaded as individual files: See our detailed guidelines for preparing your production-ready images, http://www.life-science-alliance.org/authors

-- Summary blurb (enter in submission system): A short text summarizing in a single sentence the study (max. 200 characters including spaces). This text is used in conjunction with the titles of papers, hence should be informative and complementary to the title and running title. It should describe the context and significance of the findings for a general readership; it should be written in the present tense and refer to the work in the third person. Author names should not be mentioned.

B. MANUSCRIPT ORGANIZATION AND FORMATTING:

Full guidelines are available on our Instructions for Authors page, http://www.life-science-alliance.org/authors

We encourage our authors to provide original source data, particularly uncropped/-processed electrophoretic blots and spreadsheets for the main figures of the manuscript. If you would like to add source data, we would welcome one PDF/Excel-file per figure for this information. These files will be linked online as supplementary "Source Data" files.

IMPORTANT: It is Life Science Alliance policy that if requested, original data images must be made available. Failure to provide original images upon request will result in unavoidable delays in publication. Please ensure that you have access to all original microscopy and blot data images before submitting your revision.

January 29, 2021

Re: Life Science Alliance manuscript #LSA-2020-00875R

Dr. Raghu Kalluri
University of Texas MD Anderson Cancer Center
Department of Cancer Biology
1881 East Rd
Houston, TX 77054

Dear Dr. Kalluri,

Thank you for submitting your revised manuscript entitled "Exosome-mediated delivery of CRISPR/Cas9 for targeting of oncogenic KRASG12D in pancreatic cancer" to Life Science Alliance. The manuscript has been seen by the original reviewers whose comments are appended below. While the reviewers continue to be overall positive about the work in terms of its suitability for Life Science Alliance, some important issues remain.

Our general policy is that papers are considered through only one revision cycle; however, given that the suggested changes are crucial to the main message of the paper and are addressable, we are open to one additional short round of revision.

Please submit the final revision within one month, along with a letter that includes a point by point response to the remaining reviewer comments.

To upload the revised version of your manuscript, please log in to your account:
https://lsa.msubmit.net/cgi-bin/main.plex
You will be guided to complete the submission of your revised manuscript and to fill in all necessary information.

Please pay attention to adhere to our editorial requirements for revisions:

A. THESE ITEMS ARE REQUIRED FOR REVISIONS

-- A letter addressing the reviewers' comments point by point.

-- An editable version of the final text (.DOC or .DOCX) is needed for copyediting (no PDFs).

-- High-resolution figure, supplementary figure and video files uploaded as individual files: See our detailed guidelines for preparing your production-ready images, https://www.life-science-alliance.org/authors

-- Summary blurb (enter in submission system): A short text summarizing in a single sentence the study (max. 200 characters including spaces). This text is used in conjunction with the titles of papers, hence should be informative and complementary to the title and running title. It should describe the context and significance of the findings for a general readership; it should be written in the present tense and refer to the work in the third person. Author names should not be mentioned.

B. MANUSCRIPT ORGANIZATION AND FORMATTING:

Full guidelines are available on our Instructions for Authors page, https://www.life-science-alliance.org/authors

Thank you for this interesting contribution to Life Science Alliance.

Sincerely,

Shachi Bhatt, Ph.D.
Executive Editor
Life Science Alliance
https://www.lsajournal.org/
Tweet @SciBhatt @LSAjournal

May 24, 2021

Re: Life Science Alliance manuscript #LSA-2020-00875RR

Dr. Raghu Kalluri
University of Texas MD Anderson Cancer Center
Department of Cancer Biology
1881 East Rd
Houston, TX 77054

Dear Dr. Kalluri,

Thank you for submitting your revised manuscript entitled "Exosome-mediated delivery of CRISPR/Cas9 for targeting of oncogenic KrasG12D in pancreatic cancer" to Life Science Alliance (LSA). The manuscript has been seen by the original reviewers whose comments are appended below.

As you will see from the reviewer's comments below, the reviewer is concerned about the new data that were included in the revised manuscript and points out that the reduction in KrasG12D protein level is minor. While we do appreciate the fact that you show an effect on the phosphorylation of ERK, we would like to ask you to quantify the data showing KrasG12D and pERK levels and show that it is reproducible. This would suggest that the small but significant reduction in KrasG12D has an effect. We would also request you to comment on this inefficient editing in the discussion.

This is an unusual case for LSA, as we are requesting additional data at this stage in peer-review, but given the significance of the concern raised and given that the suggested data should be easy to obtain, we are open to one additional short round of revision. Please note that we expect to make a final decision without additional reviewer input upon resubmission.

Please submit the final revision within one month, along with a letter that includes a point by point response to the remaining reviewer comments.

To upload the revised version of your manuscript, please log in to your account:
https://lsa.msubmit.net/cgi-bin/main.plex
You will be guided to complete the submission of your revised manuscript and to fill in all necessary information.

Please pay attention to adhere to our editorial requirements for revisions:

A. THESE ITEMS ARE REQUIRED FOR REVISIONS

-- A letter addressing the reviewers' comments point by point.

-- An editable version of the final text (.DOC or .DOCX) is needed for copyediting (no PDFs).

-- High-resolution figure, supplementary figure and video files uploaded as individual files: See our detailed guidelines for preparing your production-ready images, https://www.life-science-alliance.org/authors

-- Summary blurb (enter in submission system): A short text summarizing in a single sentence the study (max. 200 characters including spaces). This text is used in conjunction with the titles of papers, hence should be informative and complementary to the title and running title. It should describe the context and significance of the findings for a general readership; it should be written in the present tense and refer to the work in the third person. Author names should not be mentioned.

B. MANUSCRIPT ORGANIZATION AND FORMATTING:

Full guidelines are available on our Instructions for Authors page, https://www.life-science-alliance.org/authors

Thank you for this interesting contribution to Life Science Alliance.

Sincerely,

Shachi Bhatt, Ph.D.
Executive Editor
Life Science Alliance
http://www.lsajournal.org
Tweet @SciBhatt @LSAjournal

June 28, 2021

RE: Life Science Alliance Manuscript #LSA-2020-00875RRR

Dr. Raghu Kalluri
University of Texas MD Anderson Cancer Center
Department of Cancer Biology
1881 East Rd
Houston, TX 77054

Dear Dr. Kalluri,

Thank you for submitting your revised manuscript entitled "Exosome-mediated delivery of CRISPR/Cas9 for targeting of oncogenic KrasG12D in pancreatic cancer". We would be happy to publish your paper in Life Science Alliance pending final revisions necessary to meet our formatting guidelines.

Along with points mentioned below, please tend to the following:
-please add callouts for Figures S1 A-D, S2C, D, and S4 A-C to your main manuscript text
-please upload your supplementary figures as single files as well
-please add supplementary figure legends to the main manuscript text after the references section
-please use capital letters when introducing the panels in Figures, their legends, and their callouts
-please include Data availability statement if applicable

If you are planning a press release on your work, please inform us immediately to allow informing our production team and scheduling a release date.

To upload the final version of your manuscript, please log in to your account:
https://lsa.msubmit.net/cgi-bin/main.plex
You will be guided to complete the submission of your revised manuscript and to fill in all necessary information. Please get in touch in case you do not know or remember your login name.

To avoid unnecessary delays in the acceptance and publication of your paper, please read the following information carefully.

A. FINAL FILES:

These items are required for acceptance.

-- An editable version of the final text (.DOC or .DOCX) is needed for copyediting (no PDFs).

-- High-resolution figure, supplementary figure and video files uploaded as individual files: See our detailed guidelines for preparing your production-ready images, https://www.life-science-alliance.org/authors

-- Summary blurb (enter in submission system): A short text summarizing in a single sentence the study (max. 200 characters including spaces). This text is used in conjunction with the titles of papers, hence should be informative and complementary to the title. It should describe the context

and significance of the findings for a general readership; it should be written in the present tense and refer to the work in the third person. Author names should not be mentioned.

B. MANUSCRIPT ORGANIZATION AND FORMATTING:

Full guidelines are available on our Instructions for Authors page, https://www.life-science-alliance.org/authors

We encourage our authors to provide original source data, particularly uncropped/-processed electrophoretic blots and spreadsheets for the main figures of the manuscript. If you would like to add source data, we would welcome one PDF/Excel-file per figure for this information. These files will be linked online as supplementary "Source Data" files.

Submission of a paper that does not conform to Life Science Alliance guidelines will delay the acceptance of your manuscript.

It is Life Science Alliance policy that if requested, original data images must be made available to the editors. Failure to provide original images upon request will result in unavoidable delays in publication. Please ensure that you have access to all original data images prior to final submission.

The license to publish form must be signed before your manuscript can be sent to production. A link to the electronic license to publish form will be sent to the corresponding author only. Please take a moment to check your funder requirements.

Reviews, decision letters, and point-by-point responses associated with peer-review at Life Science Alliance will be published online, alongside the manuscript. If you do want to opt out of having the reviewer reports and your point-by-point responses displayed, please let us know immediately.

Thank you for your attention to these final processing requirements. Please revise and format the manuscript and upload materials within 7 days.

Thank you for this interesting contribution, we look forward to publishing your paper in Life Science Alliance.

Sincerely,

Eric Sawey, PhD
Executive Editor
Life Science Alliance
http://www.lsajournal.org

July 5, 2021

RE: Life Science Alliance Manuscript #LSA-2020-00875RRRR

Dr. Raghu Kalluri
University of Texas MD Anderson Cancer Center
Department of Cancer Biology
1881 East Rd
Houston, TX 77054

Dear Dr. Kalluri,

Thank you for submitting your Research Article entitled "Exosome-mediated delivery of CRISPR/Cas9 for targeting of oncogenic KrasG12D in pancreatic cancer". It is a pleasure to let you know that your manuscript is now accepted for publication in Life Science Alliance. Congratulations on this interesting work.

The final published version of your manuscript will be deposited by us to PubMed Central upon online publication.

Your manuscript will now progress through copyediting and proofing. It is journal policy that authors provide original data upon request.

Reviews, decision letters, and point-by-point responses associated with peer-review at Life Science Alliance will be published online, alongside the manuscript. If you do want to opt out of having the reviewer reports and your point-by-point responses displayed, please let us know immediately.

IMPORTANT: If you will be unreachable at any time, please provide us with the email address of an alternate author. Failure to respond to routine queries may lead to unavoidable delays in publication.

Scheduling details will be available from our production department. You will receive proofs shortly before the publication date. Only essential corrections can be made at the proof stage so if there are any minor final changes you wish to make to the manuscript, please let the journal office know now.

DISTRIBUTION OF MATERIALS:
Authors are required to distribute freely any materials used in experiments published in Life Science Alliance. Authors are encouraged to deposit materials used in their studies to the appropriate repositories for distribution to researchers.

You can contact the journal office with any questions, contact@life-science-alliance.org

Again, congratulations on a very nice paper. I hope you found the review process to be constructive and are pleased with how the manuscript was handled editorially. We look forward to future exciting submissions from your lab.

Sincerely,

Eric Sawey, PhD

Executive Editor
Life Science Alliance
http://www.lsajournal.org
